# Does Useful Field of Vision Predict Attention While Driving Between Young and Older Adults?

**DOI:** 10.1093/geroni/igab046.2771

**Published:** 2021-12-17

**Authors:** Rebecca Dunterman, R Intrieri

**Affiliations:** 1 Auburn University, Auburn, Alabama, United States; 2 Western Illinois University, Macomb, Illinois, United States

## Abstract

The Insurance Information Institute (2017) reports drivers aged 65 and up are involved in the second highest rate of fatal car crashes. It is important that there is a fair and standardized assessment to test driving fitness. The prime objective was to assess the utility of the Useful Field of View (UFOV) across young and old groups to predict performance on a simulated driving exercise. Community-dwelling adults aged 65 and older (n=48) and students (n=48) recruited from an undergraduate research pool served as participants. They completed a series of demographic, health and cognitive measures, besides a Useful Field of Vison (UFOV) task and a driving simulation exercise. Results showed that collision avoidance and braking varied between age groups, with older adults appearing to be less likely to avoid collision (Older M = 12.46, SD = 10.25, Younger (M = 7.96, SD =4.92; n = 47), but quicker to brake (Older M = 3.64, SD = 3.41, Younger M = 9.79, SD =7.91). There were group differences for driving simulator performance, predicted by cognitive measures (Young; R2 = .099, p = 0.005; Old; R2 = 0.094, p = 0.038). UFOV scores did not predict group differences in driving simulator performance (Young; R2 = 0.009, p = 0.664; β = 0.089, p = 0.437) , (Older; R2 = 0.061, p = 0.522; UFOV-DA β = -0.074, p = 0.555; UFOV_SA β = 0.289, p = 0.194). These findings have implication for the development of pragmatic capacity to drive assessments.

